# Neurological Complications Following Cardiac Surgery: Management and Future Directions

**DOI:** 10.31661/gmj.v13i.3415

**Published:** 2024-09-12

**Authors:** Sohrab Negargar, Sahar Sadeghi

**Affiliations:** ^1^ Cardiovascular Research Center of Tabriz University of Medical Sciences, Tabriz, Iran

**Keywords:** Neurological Complications, Cardiac Surgery, Stroke, Delirium, Cerebral Oximetry, ICU, MRI

## Abstract

Neurological complications related to cardiac surgery present physicians with challenges. These complications severely impact patients’ outcomes, the quality of their lives, and affect the resources available at healthcare centers. The treatment of neurological complications requires a multidisciplinary approach, where evidenced-based interventions work to identify the patient profiles and risk factors. In addition, ongoing investigations on alternative treatment methods like cerebral oximetry and personalized risk stratification may likely improve management and outcomes of such high-risk patients. Based on the significance that neurological problems play after cardiac surgery, this paper intends to offer the practitioners and scientists vital insights to help them provide the most suitable healthcare to the affected patients and to guide future studies.

## Introduction

Understanding the complex relationship between cardiac surgery and postoperative neurological problems is essential, emphasizing the importance of a thorough comprehension of this occurrence [1][2]. Complications that affect the nervous system after cardiac surgery pose a complicated and multifaceted difficulty, involving a variety of issues like strokes, confusion, mental deterioration, and nerve damage [1]. These complexities, while differing in how they appear and their causes, all have important impacts on patients’ recovery, healthcare resources, and rehabilitation. Reasons for their development range from individual patient factors like age and preexisting conditions to surgical factors such as manipulation of the aorta and the use of cardiopulmonary bypass [1][3][4][5]. The complex correlation between heart surgery and postoperative neurological issues necessitates the need to deepen the understanding of this event which accentuates the crucial role of complete comprehension [6]. The nervous system complication that occurs postoperatively after heart surgery is a very complicated and multifaceted problem, and can be subdivided into a number of issues including strokes, confusion, dementia, and nerve damage. This diversity, manifesting differently and cause-wise, all influence the patients’ recovery, the healthcare resources and the rehabilitation [1][5][7]. The causes of their development may be related to the individual patient factors such as age and preexisting conditions or to the surgical factors like aortic manipulation and cardiopulmonary bypass (CPB) [5][7].

The post-cardiac surgery neurological complications have altered the incidence of higher morbidity and mortality rate, prolonged hospitalization and expensive health care costs [6]. This review aims to help clinicians and researchers improve patient care and reduce negative neurological outcomes after cardiac surgery by explaining the reasons behind these complications and investigating successful treatment approaches.

## Etiology of Neurological Complications

**Table:** Table1. The common neurological complications following cardiac surgery, etiology, and risk factor.

**Complication**	**etiology**	**risk factors**
**Stroke**	Ischemic strokes may result from emboli originating from intracardiac sources or atherosclerotic plaques dislodged during surgery. Hemorrhagic strokes can occur due to anticoagulant therapy or vessel injury during surgical manipulation.	Advanced age, preexisting cerebrovascular disease, atrial fibrillation, carotid artery disease, and prolonged cardiopulmonary bypass time.
**Delirium**	Delirium may stem from perioperative factors such as anesthesia, sedatives, pain medications, electrolyte imbalances, or metabolic disturbances.	Older age, preexisting cognitive impairment, alcohol or substance abuse, prolonged ICU stay, and postoperative infections.
**Cognitive Decline**	Cognitive decline post-cardiac surgery can result from cerebral hypoperfusion, embolic events, inflammatory responses, or neurotoxicity associated with anesthesia or cardiopulmonary bypass.	Advanced age, preexisting cognitive impairment, history of stroke or transient ischemic attack, genetic predisposition, and perioperative cerebral hypoxia.
**Peripheral Neuropathy**	Peripheral neuropathy may arise from microvascular emboli, ischemic insults, nerve compression, or metabolic disturbances during the perioperative period.	Diabetes mellitus, peripheral vascular disease, preexisting neuropathy, prolonged surgery duration, and use of vasopressors or intraoperative hypotension.
**Seizures**	Seizures following cardiac surgery can be provoked by cerebral hypoxia, electrolyte imbalances, metabolic disturbances, or medication side effects.	Previous history of seizures, preexisting neurological conditions, perioperative hypoxemia, electrolyte abnormalities, and exposure to neurotoxic medications.

Neurological complications following cardiac surgery are based on a multitude of factors ranging from surgical-specific components of the intervention like the intrinsic technicality to patient-specific risk profiles [2][5][6].

Table-1 presented the common neurological complications following cardiac surgery [1][3][4][6]. Procedural factors include numerous parts of the very procedure: aortal handling, use of CPB, and type of anesthesia applied [6][8]. Every of such components may result in either cerebral perfusion interruptions, embolism events, or inflammatory reactions, which possibly can present a prodrome of neurologic disorders [8]. However, apart from patient’s risk factors, which are much more important in determination of the level of the patients’ susceptibility to complications, advancement of age, pre-existing vascular pathology (like severe atherosclerosis of carotid disease) and the history of stroke or stroke-like episodes are the significant predictors [3].

Elderly patients, reinforced by advanced age and concomitant physiological frailty / decreased energy reserve, are at higher risk of developing neurological complications due to compromised condition [2][5][6]. As another factor, the presence of other comorbidities such as cardiovascular disease (CVD), diabetes mellitus (DM), and hypertension increases the complexity of perioperative care and the opportunity for adverse neurological outcomes. Through the description of these factors, caregivers will be able to determine the high-risk patients as well as adopt treatment plans for perioperative management, which will serve as a way to mitigate the risk of neurological complications and hence improve patient outcomes during cardiac surgeries [1][4].

## Assessment and Diagnosis

**Table:** Table2. The medications used for the prevention and treatment of neurological complications following cardiac surgery.

**Medication**	**Action**	**Mechanism**	**Side effect**
Anticoagulants e.g. enoxaparin	Prevent and treat thromboembolic complications	Inhibit the coagulation cascade	Bleeding, heparin-induced thrombocytopenia
Anticonvulsants e.g. sodium valproate	Prevent and treat seizures	Inhibit neuronal excitability by modulating ion channels or neurotransmitter systems	Drowsiness, dizziness, ataxia, cognitive impairment
Antipsychotics e.g. Haloperidol	Manage delirium and agitation	Block dopamine and serotonin receptors in the brain	Sedation, extrapyramidal symptoms, QT prolongation, metabolic disturbances
Antiplatelet agents e.g. Aspirin	Prevent and treat ischemic stroke	Inhibit platelet aggregation and thrombus formation	Bleeding, gastrointestinal ulceration
Corticosteroids e.g. Dexamethasone	Reduce cerebral edema and inflammation	Suppress the inflammatory response and decrease vascular permeability	Hyperglycemia, infection, gastrointestinal bleeding, avascular necrosis
Antioxidants e.g. N-acetylcysteine (NAC), vitamin E	Protect neurons from oxidative stress and inflammation	Neutralize free radicals, modulate inflammatory cytokines, and prevent apoptosis	Gastrointestinal disturbances, bleeding, and increased risk of infection
Neurotrophic factors e.g. Brain-derived neurotrophic factor (BDNF), nerve growth factor (NGF)	Promote neuronal survival, differentiation, and synaptic plasticity	Enhance neurotransmitter release, modulate ion channels, and support neuronal growth	Hypotension, arrhythmias, thrombosis
N-methyl-D-aspartate (NMDA) receptor antagonists e.g. Ketamine, dextromethorphan	Reduce excitotoxicity and neuroinflammation	Block NMDA receptors, reducing glutamate-mediated neurotoxicity	Dizziness, sedation, hallucinations, and dissociative effects

Evaluation of neurological postoperative complications following the cardiac surgery has to be a sophisticated and complex one, in order to correctly show those adverse reactions. Presently, the approaches used by doctors involve a synergistic use of clinicians assessment, neuroimaging techniques and neuropsychological analysis [9][10][11]. In terms of the clinical system the intensive neurological examinations are performed on patients on a daily basis to gauge for deficits, inaccuracy, changes in mental status, and indicators for delirium or cognitive dysfunction [10].

In the clinical area of cardiac surgeries, where neuro-complications are often revealed, neuroimaging techniques are indispensable for both diagnosis and treatment planning. These digital imaging technologies, including computed tomography (CT), magnetic resonance imaging (MRI), and angiography, are consequently able to elucidate the location of brain components and their function in detail [10][11][12][13]. The recent developments in neuroimaging and the emergence of pharmacological intervention are the evidence of one of the great achievements in the management of neurological adverse effects of cardiac surgery. The research into this field has mainly concentrated on identifying neuroprotective drugs and modulating their administration and delivery regimen to effectively thwart or minimize brain damage [10].

Moreover, new imaging techniques such as functional magnetic resonance imaging (MRI) and positive emission tomography (PET) are being utilized to identify disease processes that are causing neurological impairments. Such technologies are showing promising results in detecting early signs of disease and guiding focused treatment. Not only that the advancement in imaging but also in pharmacological interventions hold great promise for better outcomes and the way forward for neuro care after surgery [12][14]. Besides, sophisticated neuroimaging methods such as discussing the automatic identification of brain lesions diffusion-weighted imaging (DWI) and angiography perfusion-weighted imaging (PWI) give more information about the viability of the tissues and the dynamics of the cerebral blood flow, guiding physicians to make right choices and to follow up the treatment. The powerful imaging technologies lead to enhanced medical care. The speedy detection of neurologic issues is one of the more significant ones. The procedures and the outcomes are better, of course [14][15][16].

Moreover, the use of neuropsychological examination for instance sensitive cognitive testing and neuropsychological evaluation is fundamental in recognizing initial cognitive differences and assessing cognitive functions overtime. The use of multiply of these assessment approaches is critical in the rapid identification of post-cardiac surgical neurological complications, selection of a particular treatment strategy and in monitoring treatment success [17][18].

The front line of the effective management of the post-cardiac surgery neurological complications lies in full multidisciplinary evaluation, which is indispensable for optimal patient care and for better outcomes. This multidisciplinary approach pools together professionals from different fields, notably, neurology, cardiology, neurosurgery, critical care, and rehabilitation for a thorough examination and treatment interventions [10][19][20].

Compiling competencies from different areas of their strengths, multidisciplinary teams can conduct comprehensive assessments, isolate etiologies, and customize individualized treatment plans based on the patient's particular situations [17][19].

Apart from that, the implementation of sophisticated imaging techniques in these assessments would contribute to a reasonable diagnostic accuracy and detect underlying possible drawbacks. It enables the provision of immediate care and treatment and, consequently, facilitating the recovery of the patient to a better state [16][19].

Incorporating a multi-disciplinary approach in the diagnostic model for healthcare providers of neurological complications leads to the improvement of diagnostic accuracy during the post-cardiac surgery period as well as the efficacy of treatment, and would ultimately improve patient outcomes [11][17].

## Management Strategies

Pharmacological Interventions

Neurological complications after cardiac surgery are best managed with pharmacological interventions as the core components of both preventive and curative therapies. Table-2 showed the common medications used for the prevention and treatment of neurological complications following cardiac surgery [9][11][21]. These interventions are a range of phases, including the pharmacological selection of the drugs targeting different physiological mechanisms underlying the neurological sequelae. Prophylactic measures are usually based on antiplatelet drugs, like aspirin or clopidogrel, used to prevent clots and reduce the risk of perioperative stroke [22].

Furthermore, anticoagulants such as heparin and low-molecular-weight heparin can be used to hinder thrombus development and improve the flow of blood in the cerebral arteries during the perioperative period [23]. The acute phase of the treatment may include the application of neuroprotective agents like neurotrophic factors, antioxidants [24] and N-methyl-D-aspartate (NMDA) receptor antagonists in order to diminish the neuronal injury and the secondary damages that can be caused due to ischemic insults [25]. On the other hand, the treatment of neuropsychiatric complications requires targeting symptoms with pharmacological management, such as the use of antipsychotics for delirium or cholinesterase inhibitors for cognitive decline [26][27].

The clinicians have the ability to reduce the incidence and severity of the adverse neurological condition by making use of the pharmacological interventions that are customized to the clinical parameters and risk factors of each patient following the cardiac surgery, thus, improving the outcome and the quality of life of the patients in the process.

Non-pharmacological Approaches

In relation with applying non-pharmacological methods monitoring the neurological complications arising after cardiac surgery one cannot overestimate the role of early detection, timely intervention and improving the quality of surgical outcomes [28][29][30]. Neurologic monitoring permanently through clinical examinations of memory, pupillary reflex and motor function assists in live evaluation of neurological status and discovery of any alterations proposing suffering disorders like stroke or delirium [26][31].

Furthermore, the use of advanced monitoring methods, such as cerebral oximetry and transcranial Doppler ultrasonography, is nothing less than a blessing, for they provide meaningful information regarding cerebral perfusion, oxygenation, and hemodynamic parameters, and with that, clinicians can identify and treat hypoperfusion or embolic events in time [11][28][32].

Through amalgamation of Non-pharmacological monitoring modalities and clinical practice, the health care providers can give exact diagnostics, can make timely interventions and can improve the outcome of treatment for the neurological complications post-cardiac surgery [30][32].

Followed by the monitoring, non-pharmacological supportive care tactics, another formidable feature of the integrative therapy when it comes to managing neurological complications post cardiac surgery [31].

This interdisciplinary team of a physician, a nurse, a physical therapist, and an occupational therapist, as well as a speech therapist, offers whole patient care individually adapted to each patient. In the first instance, early mobilizations protocols and rehabilitation programs seek to better neurological recovery, enhance physical function and avoid deconditioning [29][30].

In this respect, the employment of cognitive rehabilitation methods such as cognitive training and psychoeducation also helps to prevent cognitive deficits and promotes cognitive functions restoration. These psychosocial support services, including counseling and support groups, are aimed at addressing the emotional and psychological aspects of neurological complications [17][18]. Thus, they prove to be beneficial to both the patients and their families.

## Rehabilitation Protocols and Early Mobilization Strategies

Another key element of the protocols and motion strategies that are used during rehabilitation is the technique of avoiding the complications and encouragement of functional recovery, which also helps to prevent cognitive and language problems that may occur after cardiac surgery [33][34][35]. These severe fluxes can damage the quality of life to a serious level and limit the patient's ability to function. The incorporation of physical therapy, occupational therapy, and speech language pathology therapies in recovery protocols can significantly be a contributing factor in the treatment of cognitive and language-related disorders by helping patients regain their cognitive and language skills [36][37].

Physical therapy is essentially about strengthening the body by recovery modalities that help restore balance and increase mobility as much as possible. Therefore, activities contributed to the increase of patients' functional state and improved their general wellbeing and life satisfaction. [35] Two divergent concepts in occupational therapy are intervention approach and occupational therapy identification of patients' needs in daily living activities, cognitive function and upper extremity function. Using therapeutic techniques, occupational therapists equip the patients with the skills and strategies to take a more independent role in tackling the tasks they complete on an everyday basis [38][39]. Speech-language pathologists have the talent of the professional which is critical for the executed process of recovery by dealing with the communication difficulties, swallowing troubles, and the cognitive dysfunction [40].

Therefore, these professionals are responsible to achieve the highest level of the functional results and rehabilitate patients to enable them to convey the message properly and to participate adequately in day-to-day activities. Meanwhile, interventions of this type as well as early mobilization strategies can prevent the most common complications such as brain bleeding and blood clots. The protocols designed to reduce the risks of immobility shall include some specific exercises and activities that can successfully maintain complete blood flow and prevent muscle/bone atrophy, venous thromboembolism and pneumonia. Moreover, they can immensely help visitors recover the issue of mobility while preventing the complication of sedation such as delirium and cognitive impairment [29][34][35][39].

## Role of Cerebral Oximetry

**Figure-1:**
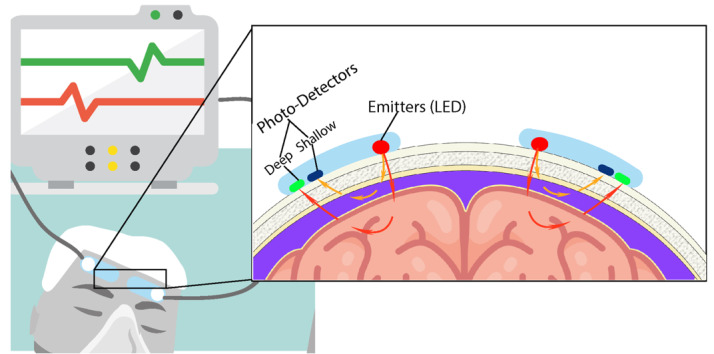
Figure 1. Schematic of an example of a cerebral oximeter sensor measuring tissue oxygenation

Cerebral oximetry is a noninvasive technique whereby regional cerebral oxygen saturation (rSO2) is measured using near-infrared spectroscopy (NIRS). The technique has been extensively used for a range of areas such as neonatology, anesthesiology, neurology, and cardiac surgery for its assessment of cerebral tissue oxygenation (Figure-1) [41].

Brain oximetry is most useful during critical times to ensure adequate oxygenation supply, such as the passage from fetal to neonatal life in cases of preterm infants, cardiopulmonary resuscitation (CPR), and during anesthesia and surgery period [41][42].

The applications of cerebral oximetry in the last few years have provided evidence that could be used to reduce the neurological issues after cardiac surgery. Cerebral oximetry provides continuous monitoring of cerebral oxygenation in real time performance that helps detect any hypoxic episodes before their onset [32][43][44].

Through the use of cerebral oximetry in perioperative care, healthcare providers will enjoy an opportunity to identify and deal with any alterations in cerebral perfusion, thereby diminishing chances of postoperative neurological adverse effects [44].

The future of postoperative neurological care is likely to be in finding ways to further improve the use of cerebral oximetry in perioperative routines, exploring what the later long-term neurological outcomes are for people with oximetry, and discovering which patient populations benefit the most from this technology.

Expectantly, with the progress of the field, a more complete knowledge of cerebral oximetry for improving acute complications after cardiac surgery would be the solid basis for evidence-based management strategies of neurological complications post cardiac surgery.

## Emerging Research Trends and Future Directions

Current research corridors of post-surgical neurological problems treatment encompass several directions with a good prospect to be implemented in clinical practice and to raise patients’ survival prognosis. Integration of advanced neuroimaging modalities including functional MRI and diffusion tensor imaging is one of the trends of research that provide a deeper perspective of neuropathology and discover biomarkers useful for risk stratification and prognostication [16]. Furthermore, the development of specific neuroprotective substances and medications that interfere with the process of ischemia and inflammation by targeting certain pathways show great promise for reducing the patients' injury and promoting the post-operative neurological recovery [24].

Moreover, the tendency for the utilization of modern perioperative monitoring technologies, among which are continuous an electroencephalogram ( EEG) monitoring and near-infrared spectroscopy, opens up the chances for the real-time analysis of cerebral function and perfusion, thus, effects on the early detection and intervention affects those patients that are at the risk of neurological complications [41][45][46]. Similarly, the development of multidisciplinary care plans involving neurologists, intensivists, and rehabilitation experts who can take a holistic approach to care as well as consider patients' comorbidities can be a good way to meet patients' needs [18][21][39].

Ultimately, the emergence of precision medicine technology that relies on genetic profiling and personalized risk stratification algorithms for the identification of individuals with high likelihood of adverse neuropsychological consequences and to direct specific interventions aimed at minimizing the side-effects in this patient population [47]. Through wrapping up the aforementioned up- and -coming research trends as well as future directions, clinicians and researchers will be in a good position to further curb the complications of the said neurological disorders which might occur after cardiac surgery.

## Conclusion

This review has synthesized key findings regarding the management of neurological complications following cardiac surgery, highlighting the multifactorial nature of these events and the diverse approaches to their prevention and treatment. Evidence supports the efficacy of pharmacological interventions, non-pharmacological strategies, and advanced monitoring techniques, such as cerebral oximetry, in reducing the incidence and severity of complications. Furthermore, emerging research trends, including the integration of advanced neuroimaging, targeted neuroprotective agents, and precision medicine approaches, hold promise for further optimizing patient outcomes. The implications for clinical practice underscore the importance of a multidisciplinary approach, individualized patient care, and proactive management strategies tailored to specific risk profiles. Future research directions should focus on refining risk stratification models, elucidating underlying pathophysiological mechanisms, and evaluating novel interventions to enhance the prevention and management of neurological complications post-cardiac surgery. By addressing these challenges and opportunities, clinicians and researchers can advance our understanding and management of neurological complications, ultimately improving patient care and outcomes in this vulnerable population.

## Conflict of Interest

None declared.
